# [^18^F]FDG PET/CT predicts progression-free survival in patients with idiopathic pulmonary fibrosis

**DOI:** 10.1186/s12931-017-0556-3

**Published:** 2017-04-27

**Authors:** Aurélien Justet, Astrid Laurent-Bellue, Gabriel Thabut, Arnaud Dieudonné, Marie-Pierre Debray, Raphael Borie, Michel Aubier, Rachida Lebtahi, Bruno Crestani

**Affiliations:** 1 APHP, Hôpital Bichat, Service de Pneumologie A, DHU FIRE, Centre de compétence des maladies pulmonaires rares, 46 rue Henri Huchard, 75018 Paris, France; 2 APHP, Hôpital Beaujon Service de Médecine nucléaire, Clichy, France; 3 APHP, Hôpital Bichat, Service de Pneumologie et de Transplantation Pulmonaire, DHU FIRE, Paris, France; 4 APHP Hôpital Bichat, Service de Radiologie, Paris, France; 5 Université Paris Diderot, Sorbonne Paris Cité, Paris, France

**Keywords:** Pulmonary fibrosis, PET scan, Total lesion glycolysis, Prognosis

## Abstract

**Background:**

Idiopathic pulmonary fibrosis (IPF) is a devastating disease characterized by an unpredictable course. Prognostic markers and disease activity markers are needed. The purpose of this single-center retrospective study was to evaluate the prognostic value of lung fluorodeoxyglucose ([^18^F]-FDG) uptake assessed by standardized uptake value (SUV), metabolic lung volume (MLV) and total lesion glycolysis (TLG) in patients with IPF.

**Methods:**

We included 27 IPF patients (IPF group) and 15 patients with a gastrointestinal neuroendocrine tumor without thoracic involvement (control group). We quantified lung SUV mean and SUV max, MLV and TLG and assessed clinical data, high-resolution CT (HRCT) fibrosis and ground-glass score; lung function; gender, age, physiology (GAP) stage at inclusion and during follow-up; and survival.

**Results:**

Lung SUV mean and SUV max were higher in IPF patients than controls (*p* <0.00001). For patients with IPF, SUV mean, SUV max, MLV and TLG were correlated with severity of lung involvement as measured by a decline in forced vital capacity (FVC) and diffusing capacity of the lungs for carbon monoxide (DLCO) and increased GAP score. In a univariate and in a multivariate Cox proportional-hazards model, risk of death was increased although not significantly with high SUV mean. On univariate analysis, risk of death was significantly associated with high TLG and MLV, which disappeared after adjustment functional variables or GAP index. Increased MLV and TLG were independent predictors of death or disease progression during the 12 months after PET scan completion (for every 100-point increase in TLG, hazard ratio [HR]: 1.11 (95% CI 1.06; 1.36), *p* = 0.003; for every 100-point increase in MLV, HR: 1.20 (1.04; 1.19), *p* = 0.002). On multivariable analysis including TLG or MLV with age, FVC, and DLCO or GAP index, TLG and MLV remained associated with progression-free survival (HR: 1.1 [1.03; 1.22], *p* = 0.01; and 1.13 [1.0; 1.2], *p* = 0.005).

**Conclusion:**

FDG lung uptake may be a marker of IPF severity and predict progression-free survival for patients with IPF.

## Background

Idiopathic pulmonary fibrosis (IPF) is a rare and devastating disease characterized by an exaggerated accumulation of extracellular matrix and fibroblasts leading to destroyed alveoli [1] and to death within 5 years of diagnosis in most patients [2]. Advances in management are hampered by the heterogeneity of disease progression. Currently, the main prognostic factors are forced vital capacity (FVC) and diffusing capacity of the lungs for carbon monoxide (DLCO) at diagnosis and the decline of FVC and/or DLCO during 6 or 12 months [3, 4]. The decline in 6-min walk test (6MWT) distance can also predict survival [5]. Novel methods of IPF staging include the gender, age, and physiology (GAP) index and staging system, which predicts survival [6]. Many biomarkers are of potential interest as prognostic factors but none is currently used in daily practice [7–9].

Recent studies identified a metabolic shift in IPF with increased glycolysis, particularly in fibroblasts [10, 11], a finding also confirmed by microarray studies of IPF lung tissue [12]. Positron emission tomography (PET) offers the ability to non-invasively investigate cellular glucose metabolism in vivo. Recently, the fluorodeoxyglucose ([^18^F]FDG) PET signal was found consistently increased and objectively measurable in patients with pulmonary fibrosis [13]. Specifically, increased radionuclide uptake occurs in areas of honeycombing, which suggests that these fibrosis regions may be more biologically active than previously believed [13]. Two studies, involving a total of 26 patients with IPF, found a negative correlation between lung [^18^F]FDG uptake, assessed by standardized uptake value (SUV), and lung function [13, 14]. Other metrics of metabolic activities include metabolic lung volume (MLV) and total lesion glycolysis (TLG). TLG is the product of the MLV and SUV mean [15]. TLG could be better than SUV mean or SUV max as a prognostic marker because it accounts for intracellular glucose accumulation within the total volume of all regions of interest [16]. Recently, TLG was found to predict overall survival in patients with liver colorectal metastases and those with lung cancer treated with erlotinib [16, 17]. Recently, one study demonstrated the prognostic value of dual-time-point [^18^F]FDG PET for IPF [18]. Despite a better prognostic value than SUV, no study has assessed the different metric metabolic activities and evaluated the prognostic impact of [^18^F]FDG PET signal measured by MLV and TLG in IPF. We aimed to assess lung [^18^F]FDG uptake, measured by SUV mean, SUV max, MLV and TLG, as a prognostic factor for IPF.

## Methods

This study was conducted in accordance with the amended Declaration of Helsinki. The protocol was approved by the Institutional Review Board of the French society for respiratory medicine (Société de Pneumologie de Langue Française; CEPRO 2012-016), and written informed consent was obtained from all patients.

### Patient selection

We asked all consecutive patients with a diagnosis of IPF, with a typical usual interstitial pattern (UIP) on high resolution CT of the chest (HRCT), to undergo [^18^F]FDG PET. According to ATS/ERS/JRS/ALAT guidelines, a typical UIP pattern was defined as the combination of reticular abnormalities, honeycombing with or without traction bronchiectasis, predominantly localized in sub-pleural areas with absence of features listed as inconsistent [19]. Patients receiving oral corticosteroids, immunosuppressants, or any antifibrotic therapy (including pirfenidone and nintedanib) within 3 months before PET completion were excluded. All patients received a diagnosis after a multidisciplinary discussion involving review of all clinical, functional, imaging and pathological data, according to French and international guidelines [19, 20]. Former smokers were defined by smoking cessation for at least 6 months.

### Data analysis

For all patients, we assessed clinical data, bronchoalveolar lavage (BAL) cytology results, pulmonary function test results, and 6MWT distance performed within 1 month after PET. We applied the GAP index and staging system to each patient to obtain the GAP stage according to Ley et al. [6].

HRCT was obtained within 2 months after PET evaluation. The extent of fibrosis and ground glass was quantified for each lung lobe according to Kazerooni et al. [21]. The fibrosis score and ground-glass score for the left and right lung was calculated as the sum of the scores for each lobe. The total lung score was calculated as the geometric mean of the left and right lung scores. Duration of follow-up was defined as the time between PET completion and the date of the latest news, transplantation or death. During follow-up, we analyzed the changes in FVC and DLCO during the 12 months after PET completion. Acute exacerbation, defined according to Collard et al [22], was recorded. In further analysis, disease progression was defined as all-cause mortality, acute exacerbation, 10% or more decline in absolute FVC value at 12 months, or 15% or more decline in absolute DLCO value at 12 months, according to Collard et al. [4].

### Controls

The control group included patients with gastrointestinal neuroendocrine tumor without thoracic localization who underwent [^18^F]FDG PET for pre-therapeutic evaluation of the tumor. None of these patients had evidence of current or past respiratory disease or had received thoracic radiotherapy or systemic corticosteroids. None had parenchymal abnormalities on chest CT coupled with PET.

### PET analysis

**Fig. 1:**
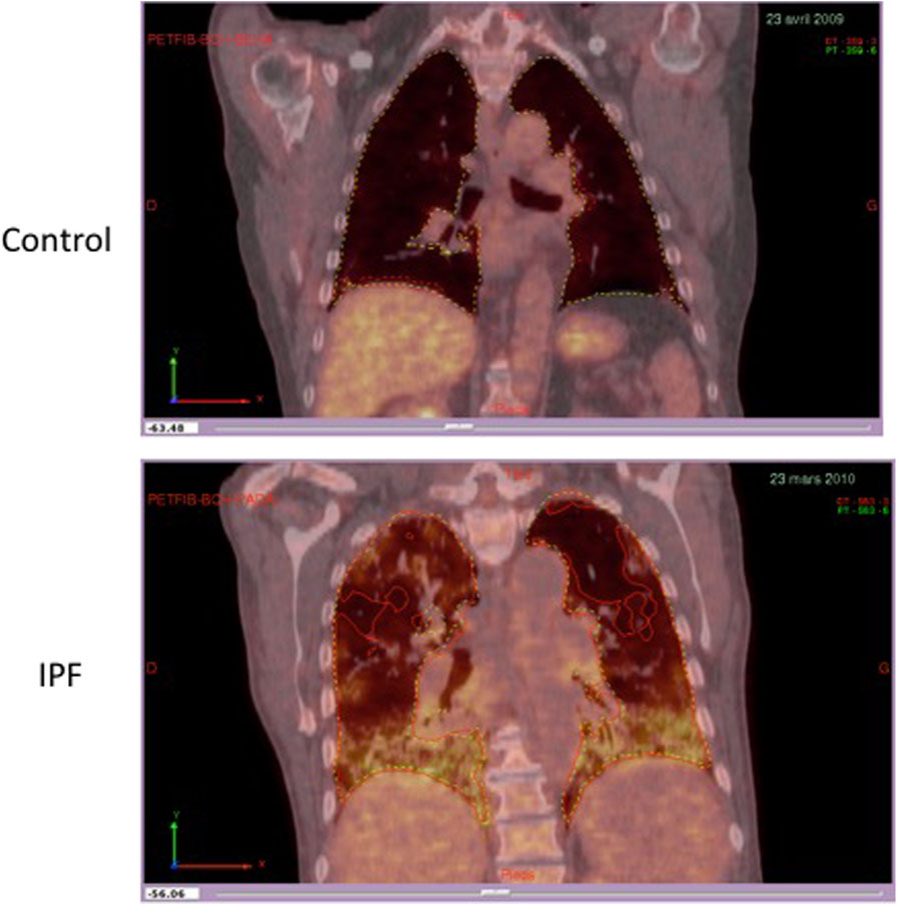
Representative lung regions of interest for analysis in control patients (at the *top*) and in IPF patients (at the *bottom*). The area in *yellow* represents the lung surface. The area in *red* represents the metabolic lung volume

[^18^F]FDG PET/CT involved a PET-CT scanner (GE Discovery 690, GE Health-care, Milwaukee, WI, USA). All patients fasted for at least 6 h before the examination. The serum glucose levels were < 9 mmol/L. PET-CT images were obtained 60 min after injection of 4 MBq/kg [^18^F]FDG. We performed iterative construction with attenuation correction, scatter correction, variant point spread function compensation and time of flight. The time of the image acquisition at 60 min was 3 min per bed. The regions of interest were manually identified. A visual analysis described the regional distribution of FDG uptake. Quantitative analysis was then performed. The SUV mean and SUV max were obtained for the right and left lung. Hereafter, the SUV mean is the geometric mean of SUV mean for the left and right lungs and the SUV max is the geometric mean of SUV max for the left and right lungs. The whole lung was delineated by an automatic lung extraction available in Planet software (DOSIsoft, Cachan, France) (Fig. 1, outlined yellow area). The bronchi were not excluded. The uptake from liver mediastinum (including heart but excepting adenopathy) was manually excluded when needed. Figure 1 shows the representative regions of interest used for analysis in a control patient (at the top) and in IPF patient (at the bottom). The area outlined in yellow represents the lung volume. The area outlined in red represents the metabolic lung volume. The MLV was determined considering a threshold of 1, considering the normal lung SUV mean to be 0.5. This was determined by measuring the mean SUV in lung of normal patients. We then applied a safety margin by using a threshold of 1. Therefore, we ensured that the MLV contained no or very few normal lung voxels. The relevant PET-related variables were extracted for the MLV and the whole lung. The variables were the maximum SUV (SUV max) and average SUV (SUV mean), from which we derived the TLG [ie, the product of the MLV and SUV mean [23]].

### Statistical analysis

Statistical analysis involved use of GraphPad v6 and R v2.15. Because the data followed a normal distribution, we used parametric tests for analysis. Student *t* test was used to compare data for two groups and Pearson correlation test to determine correlation between two variables. Survival was estimated by the Kaplan-Meier survival curves and compared by log-rank test. Univariate and multivariate Cox proportional-hazards models were used to analyze the relationship between the variables of interest and survival and progression-free survival, estimating hazard ratios (HRs) and 95% confidence intervals (CIs). Because SUV, TLG and MLV were highly correlated (r > 0.9), they were not included together in the multivariate models. *P* < 0.05 was considered statistically significant.

## Results

We included 27 patients with IPF (mean age 65.3 ± 12.4 years) (Table 1): 11 were never smokers, four were current smokers and 12 were former smokers. The mean duration of IPF since diagnosis was 2.9 ± 2.7 years at the time of PET scan completion. Mean follow-up was 1.6 ± 0.9 years after PET scan. At the time of the PET scan, seven patients (26%) were in GAP stage I, 12 (44%) in GAP stage II and 8 (30%) in GAP stage III. Mortality during follow-up was 44% (*n* = 12), with a median survival of 839 days. The main cause of death (*n* = 11) was respiratory failure. Four patients experienced an acute exacerbation during follow-up. Lung cancer developed in one patient 2 years after evaluation. Follow-up evaluation of pulmonary function tests was available for 22 patients; three were lost to follow-up and two died before the completion of follow-up evaluation.

**Table 1:** Clinical and functional data for patients (*n* = 27) with interstitial pulmonary fibrosis (IPF)

Characteristics	
Age (years)	65.3 ± 12.4
Duration of disease (years)	2.9 ± 2.7
Sex ratio (M/F), no.	22/5
Mortality (%)	44% (12/27)
Survival (years)	1.5 ± 1.2
Duration of follow-up (years)	3.4 ± 1.2
Smoking status, n (pack-years)	
Current smoker	4 (45 ± 5)
Former smoker	12 (27 ± 12)
Never smoker	11
Lung function tests	
FVC (L)	2.3 ± 0.9
FVC (% predicted)	71 ± 26
DLCO (% predicted)	41 ± 16
Distance traveled during 6MWT (meters) (meters) (meters)=21)	457 ± 76
PaO2 (mmHg)	69.2 ± 9.1
PaCO2 (mmHg)	36.8 ± 2.6
Bronchoalveolar lavage cytology (*n* = 19) Cellularity (x103/mL)	263 ± 19
Macrophages (%)	62.0 ± 4.6
Lymphocytes (%)	9.3 ± 1.6
Neutrophils (%)	22.2 ± 5.0
Eosinophils (%)	2.5 ± 1.6

Data are mean ± SD unless indicated

The mean delay between chest HRCT and PET was 5.2 ± 2.6 weeks. HRCT showed a typical UIP pattern in all patients as per the inclusion criteria. The mean fibrosis score was 8.2 ± 3.0 and mean ground-glass score 2.8 ± 1.9 (Table 2). As expected, the fibrosis score was higher in lower than upper lobes.

**Table 2:** Lung [^18^F]FDG PET and high-resolution CT (HRCT) analysis in IPF patients

	Controls		IPF patients		
	Global	Global	Right lung	Left lung	*P* value
[^18^F]FDG PET analysis					
SUV max	1.84 ± 0.82	3.8 ± 2.5	3.7 ± 2.5	3.9 ± 2.5	0.45
SUV mean	0.50 ± 0.11	1.0 ± 0.4	1.0 ± 0.5	1.0 ± 0.3	0.57
TLG (cm^3^)	0	1426 ± 1030			
MLV (cm^3^)	0	950 ± 571			
HRCT analysis					
Fibrosis score	NA	8.2 ± 3.0	8.4 ± 3.1	7.9 ± 3.0	0.86
Ground-glass score	NA	2.8 ± 1.9	2.6 ± 1.9	3.0 ± 2.0	0.65

Data are mean ± SD. In controls, HRCT was not performed and global [^18^F]FDG uptake was quantified. NA: data not available

Overall, 15 patients (nine men) were included in the control group. Mean age was 54.5 ± 12.2 years; 60% were former smokers.

### PET analysis

In the control group, the lung SUV mean was 0.50 ± 0.11 and SUV max was 1.84 ± 0.82. SUV mean and SUV max did not differ between the left and right lungs. Because of the lack of hypermetabolic volume in controls, the TLG value was null.

**Fig. 2:**
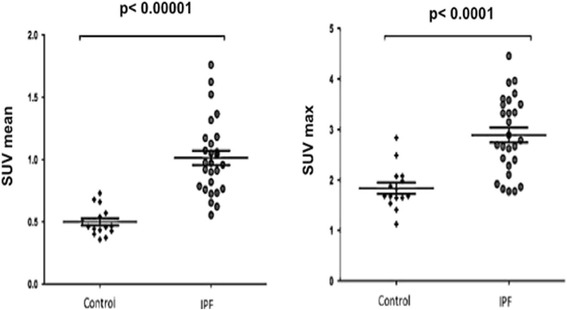
Comparison of SUV between IPF patients and controls

All IPF patients showed increased [^18^F]FDG uptake as compared with controls, both in the quantitative and visual analysis. SUV mean and SUV max were higher for IPF patients than controls (1.0 ± 0.4, *p* < 0.00001, and 3.8 ± 2.5, *p* < 0.0001, respectively) (Fig. 2). SUV mean and SUV max did not differ between the right and left lungs (Table 2). The mean TLG was 1426 ± 1030 cm^3^ and mean MLV 950 ± 571 cm^3^. Increased pulmonary [^18^F]FDG uptake was predominant in sub-pleural areas and corresponded to honeycombing/reticulation areas.

### [^18^F]FDG uptake correlated with more severe disease

**Fig. 3:**
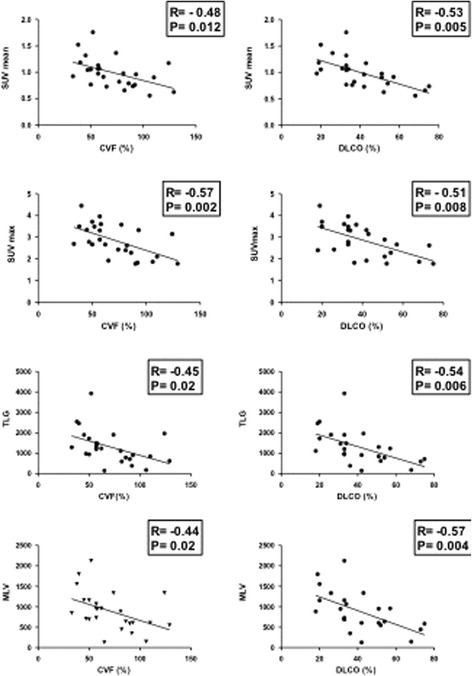
Correlation between lung [^18^F]FDG uptake and pulmonary function test results

**Fig. 4:**
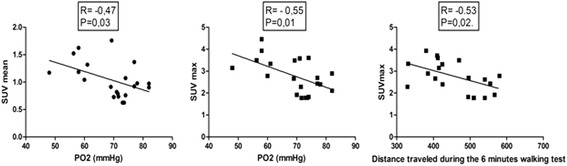
Correlation between lung [^18^F]FDG uptake and PaO_2_ and distance traveled during the 6-min walk test

**Fig. 5:**
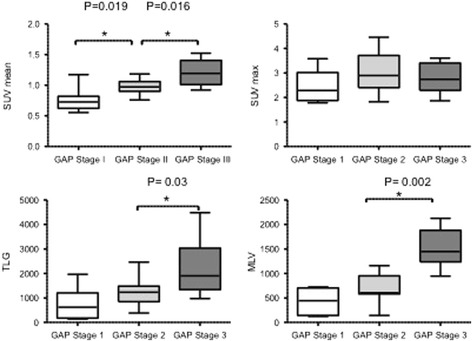
Comparison of SUV mean, TLG and MLV by GAP score

We found no significant correlation between SUV mean, SUV max, MLV or TLG and epidemiological data, BAL cytology analysis or extent of fibrosis indicated by HRCT fibrosis and ground-glass scores. By contrast, we found a negative correlation between SUV mean, SUV max, MLV, TLG and lung function test results for FVC, DLCO, force expiratory volume in 1 s and total lung capacity (expressed in liters or as a percentage predicted value), which indicates increased uptake of [^18^F]FDG correlated with more severe disease (Fig. 3). We also found a negative correlation between partial pressure of oxygen (PaO2) and SUV mean and SUV max (Fig. 3). Furthermore, SUV max was negatively correlated with 6MWT distance, with no correlation between SUV mean or TLG and this variable (Fig. 4). High GAP stage was associated with high SUV mean, MLV and TLG (Fig. 5).

SUV mean, SUV max, MLV or TLG did not differ between former or active smokers and non-smokers and were not correlated with pack-years.

### Lung function decline

Follow-up results of pulmonary function tests were available for 22 patients: the mean FVC decline was 290 mL ± 251 mL (8.4% ± 8.0%, absolute value) and mean DLCO decline 10.1% ± 8.1% (absolute value). We found no correlation between the FVC and DLCO decline and SUV mean, SUV max, MLV or TLG. We then assessed [^18^F]FDG uptake by disease progression (Table 3), defined as all-cause mortality, acute exacerbation, 10% or more decline in FVC (absolute value) at 12 months, or 15% or more decline in DLCO (absolute value) at 12 months. In all, 12 patients (44%) were considered to have disease progression. TLG and MLV were significantly higher in patients with than without disease progression (Table 3). Similarly, TLG and MLV were higher for non-survivors than survivors during follow-up. SUV mean and SUV max did not differ between patients with and without disease progression and survivors and non-survivors (Table 3).

**Table 3:** Lung [^18^F] FDG uptake analysis by survival and disease progression

	Survivor	Non-survivor	*P* value	Disease progression	No disease progression	*P* value
SUV max	3.9 ± 0.2	3.4 ± 0.2	0.2	3.7 ± 0.2	3.3 ± 0.4	0.1
SUV mean	1.1 ± 0.1	0.9 ± 0.1	0.4	1.0 ± 0.1	0.8 ± 0.1	0.3
TLG (cm^3^)	873.7 ± 184.9	1783 ± 230.6	0.005	1711 ± 149.5	945.3 ± 189.8	0.02
MLV (cm^3^)	784.6 ± 175.6	1331.2 ± 154.0	0.029	657.7 ± 115.5	1307 ± 159.2	0.003

Data are mean ± SD

### Survival analysis

On univariate analysis, risk of death was increased for patients with FVC or DLCO decline and those with increased TLG or MLV; risk of death was increased with high SUV mean but not significantly (Table 4). On multivariate analysis, FVC (likelihood ratio test = 7.03, *p* = 0.02) and DLCO (likelihood ratio test = 6.58, *p* = 0.04) were the only independent factors associated with survival.

**Table 4:** Univariate and multivariate logistic regression analysis of variables associated with survival for IPF patients

	HR (95% CI)	*P* value
Univariate analysis		
TLG (increasing 100 cm^3^)	1.08 (1.01; 1.16;)	0.04
MLV (increasing 100 cm^3^)	1.13 (1.01; 1.26;)	0.04
SUV mean (increasing 0.1 unit)	1.23 (1.00; 1.52)	0.058
SUV max (increasing 0.1 unit)	1.05 (0.96; 1.15)	0.27
FVC (increasing 10%)	0.73 (0.54; 0.97)	0.01
DLCO (increasing 10%)	0.6 (0.39; 0.92)	0.01
Fibrosis CT score (increasing 1 unit)	1.15 (0.91; 1.44)	0.24
Ground-glass CT score (increasing 1 unit)	0.46 (0.16; 1.37)	0.12
Multivariate analysis		
TLG (including FVC and DLCO)	1.05 (0.96; 1.15)	0.26
TLG (including GAP index)	1.08 (1.0; 1.17)	0.06
MLV (including FVC and DLCO)	1.07 (0.93; 1.23)	0.38
MLV (including GAP index)	1.13 (0.98; 1.30)	0.09

### Progression-free survival in the 12 months after PET completion

On univariate analysis, risk of death or disease progression was increased for patients with high SUV mean, TLG or MLV during the 12 months after PET completion (Table 5). In a multivariable model including SUV mean, TLG or MLV together with age, FVC and DLCO, risk of death or disease progression remained increased with high TLG and MLV (Table 5) even when the GAP index was included in the model (Table 5).

**Table 5:** Univariate and multivariate logistic regression analysis of variables associated with progression-free survival for IPF patients

	HR (95% CI)	*P* value
Univariate analysis		
TLG (increasing 100 cm^3^)	1.11 (1.06; 1.36)	0.003
MLV (increasing 100 cm^3^)	1.20 (1.04; 1.19)	0.002
SUV mean (increasing 0.1 unit)	1.28 (1.06; 1.55)	0.01
SUV max (increasing 0.1 unit)	1.08 (0.99; 1.16)	0.06
FVC (increasing 10%)	0.86 (0.69; 1.06)	0.14
DLCO (increasing 10%)	0.84 (0.61; 1.17)	0.30
Multivariate analysis		
TLG (including FVC and DLCO)	1.22 (1.03; 1.22)	0.01
TLG (including GAP index)	1.13 [1.09; 1.24)	0.005
MLV (including FVC and DLCO)	1.23 (1.05; 1.45)	0.01
MLV (including GAP index)	1.27 (1.09; 1.47)	0.005

*HR* hazard ratio; 95% CI, 95% confidence interval

**Fig. 6:**
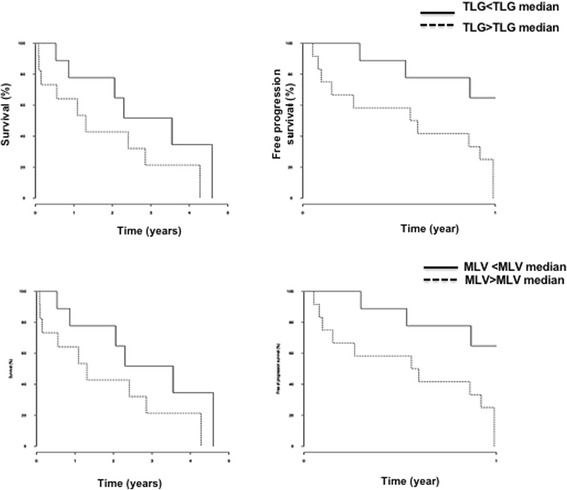
Kaplan-Meier curves for survival by median TLG and MLV values

When the cohort of patients was divided by median TLG value (1218 cm^3^), progression-free survival was better for patients with TLG values below than above the threshold (367 days [317 - ∞] vs 211 [55 - ∞]; *p* = 0.02) (Fig. 6). We observed the same result when the cohort was divided by median MLV (862 cm^3^). Progression-free survival was better for patients with MLV values below than above the threshold (367 days [317 - ∞] vs 211 days [55 - ∞]; *p* = 0.02).

## Discussion

To our knowledge, this is the second largest series of patients with IPF involving study with [^18^F]FDG PET and the first study to assess the usefulness of TLG and MLV in assessing IPF prognosis. [^18^F]FDG uptake was higher for IPF patients than controls (Fig. 1) and [^18^F]FDG uptake was positively correlated with disease severity (assessed by multiple indexes of lung function alteration and GAP stage). Furthermore, MLV and TLG but not SUV mean or SUV max were independent factors of progression-free survival.

Our results are consistent with previous experimental and clinical studies. In bleomycin-induced pulmonary fibrosis in mice, lung mean SUV was correlated with histologic score of fibrosis, and lung hydroxyproline content strongly suggested that [^18^F]FDG uptake was related to the fibrotic process [24]. In a human study, [^18^F]FDG uptake (SUV max) was correlated with FVC and DLCO [13]. Recently, in a small study involving 8 IPF patients, SUV mean was correlated with FVC at first evaluation and FVC decline and increased SUV max at repeated PET analysis [11]. Assessing a higher number of patients, we found a marginally significant correlation between FVC and SUV mean or SUV max and no correlation with FVC decline. Despite a clear correlation between [^18^F]FDG uptake and altered lung function, we found no correlation between [^18^F]FDG uptake and fibrosis extent assessed by the HRCT fibrosis score, which might be due to the use of the Kazerooni score [21]. This fibrosis score has been shown to correlate well with the histopathology fibrosis score, whereas the ground-glass score did not correlate well with the inflammation histopathology score, so ground-glass likely represents fibrosis in part [14]. Recently, Umeda and coworkers used the retention index of standardized uptake value (RI-SUV), calculated from dual-time-point [^18^F]FDG PET early (1-h conventional scan) and delayed (2–3 h) imaging findings in a series of 50 IPF patients [13]: positive RI-SUV strongly predicted early deterioration of pulmonary function and high mortality in patients with IPF. These results must be confirmed [18].

Our study showed that TLG and MLV but not SUV were independent prognostic factors in IPF. SUV mean was associated with prognosis in our population but did not reach statistical significance. We found a strong correlation between MLV and SUV mean (r = 0.90); this result is probably due to the few patients included in the study. Our result is consistent with observations in oncology, with some studies suggesting TLG as a better predictor of outcomes in patients with lung or cervical cancer [17, 25]. We found MLV independently associated with prognosis, like TLG, so the volume of the lung with an altered uptake may be more informative to assess prognosis than the mean SUV in this volume. Indeed, TLG incorporates metabolic volume and SUV mean and theoretically assesses the total activity of all metabolically active cells, whereas SUV mean assesses the activity of metabolically and non-metabolically active cells in the lung.

The methodological limitations of our study are mainly related to its single-center design and the inclusion of a relatively small number of patients, although this is the second-largest series published. Moreover, our results cannot be generalized to the whole population of patients with IPF. We excluded patients with a possible UIP pattern in order to minimize the risk of including patients with an alternative diagnosis. In the control group, we included only patients with a gastrointestinal neuroendocrine tumor without thoracic localization in order to limit a SUV bias measurement linked to the cancer. Concerning the SUV uptake assessment, we limited this study to the quantification of the SUV mean and SUV max per lung. Other methods — a 3D definition of the region of interest or a semi-automatic 2D analysis [26] — have been used to quantify SUV max. Those methods would probably have allowed for a better description of the uptake in fibrotic areas.

Beside [^18^F]FDG uptake, attempts have been made to monitor lung fibrosis with nuclear medicine markers. Gallium (67) uptake is increased in IPF, does not correlate with lung function test results and HRCT fibrosis scores, and increases over time in patients with IPF [27]. Our group previously showed increased octreotide uptake in the IPF lung and correlated with altered lung function and HRCT fibrosis score [28]. Lung function tests (FVC, DLCO) are well-validated, non-invasive, highly standardized and relatively cheap tools to assess IPF severity and prognosis. However, we still need better tools to evaluate prognosis and response to antifibrotic therapies. Metabolic imaging, such as [^18^F]FDG uptake, may have potential to fulfill these tasks in the future.

Increased [^18^F]FDG uptake is a marker of aerobic glycolysis in cancer cells [29]. Aerobic glycolysis was recently found increased in the IPF lung [10, 11]. Our data suggest that glucose metabolism reflects at least in part the remodeling and fibrotic process in the lung. Identification of the cells responsible for [^18^F]FDG uptake in the fibrotic lung is tricky. [^18^F]FDG is a glucose analogue whose intracellular transport is under glucose transporter 1 (GLUT1) dependence. Alveolar type two pneumocytes, fibroblasts, and bronchial epithelial cells express GLUT1 [30–34], although one study suggested that GLUT1 was expressed by erythrocytes and inflammatory cells but not epithelial cells and fibroblasts [35]. We found no correlation between BAL cytology and SUV mean, SUV max, MLV and TLG, supporting the idea that inflammatory cells are not the main source for lung FDG uptake in patients. Interestingly, one recent study confirmed the expression of GLUT1 by lung fibroblast in mice and showed that GLUT-1 inhibition significantly inhibited bleomycin-induced lung fibrosis in mice, which offers new opportunities for IPF treatment [34].

## Conclusions

Our study clearly establishes that lung [^18^F]FDG uptake in IPF is strongly linked to the severity of altered lung function. Lung [^18^F]FDG uptake assessed by MLV and TLG is an independent predictor of progression-free survival during the 12 months after PET. Further studies are needed to determine the usefulness of [^18^F]FDG PET in the clinical management of patients with fibrotic lung diseases.

## Abbreviations

6 MWT: 6-min walk test
BAL: Bronchoalveolar lavage
DLCO: Diffusion capacity of carbon monoxide
FVC: Forced vital capacity
HRCT: High-resolution CT
IPF: Idiopathic pulmonary fibrosis
MLV: Metabolic lung volume
SUV: Standardized uptake value
TLG: Total lesion glycolysis
UIP: Usual interstitial pneumonitis
